# Recent Approaches for Bioactive Peptides Production from Pulses and Pseudocereals

**DOI:** 10.3390/molecules30214304

**Published:** 2025-11-05

**Authors:** Manuel Martoccia, Vincenzo Disca, Yassine Jaouhari, Matteo Bordiga, Jean Daniel Coïsson

**Affiliations:** Department of Pharmaceutical Sciences, Università degli Studi del Piemonte Orientale “Amedeo Avogadro”, Largo Donegani 2, 28100 Novara, Italy; manuel.martoccia@uniupo.it (M.M.); vincenzo.disca@uniupo.it (V.D.); yassine.jaouhari@uniupo.it (Y.J.); jeandaniel.coisson@uniupo.it (J.D.C.)

**Keywords:** bioactive peptides, pulses, pseudocereals, bioactivity, bio-based approaches

## Abstract

Pulses and pseudocereals are sustainable protein sources of bioactive peptides (BAPs) with potential antioxidant, antihypertensive, antidiabetic, antimicrobial, and immunomodulatory activities. BAPs are typically liberated during gastrointestinal digestion or through bio-based processes, among which enzymatic hydrolysis and microbial fermentation represent the most widely applied strategies. Enzymatic hydrolysis provides controlled and reproducible release of short peptide motifs; recent advances such as ultrasound- or high-pressure–assisted hydrolysis enhance yield and bioactivity. Fermentation exploits microbial proteolytic activity to generate complex peptide mixtures, while improving sensory quality, reducing antinutritional compounds, and responding to consumer demand for natural and “clean-label” products. *In silico* tools increasingly complement these approaches by accelerating peptide discovery, predicting interactions with molecular targets, and guiding process design. This review provides an updated overview of bio-based methods to produce BAPs from pulses and pseudocereals, emphasizing the comparative advantages of enzymatic and fermentation technologies and their integration with computational tools. Moreover, it examines regulatory frameworks in the European Union, the United States, Japan, and China, while discussing current challenges for industrial scale-up and application in functional foods and nutraceuticals. These combined strategies offer a promising pathway to unlock the health and sustainability potential of plant proteins.

## 1. Introduction

### 1.1. Rationale and Background

Bioactive peptides (BAPs) are short amino acid sequences encrypted within food proteins that, once released, can exert a wide range of physiological effects, including potential antioxidant, antihypertensive, antidiabetic, antimicrobial, prebiotic and immunomodulatory activities. Over the last decades, dairy and soy proteins have been the primary focus of peptide research, owing to their abundance and well-characterized bioactivity. However, with the rising global demand for sustainable food systems and healthier dietary options, there is increasing interest in identifying alternative plant-based protein sources for BAP production. At the same time, the growing incidence of chronic metabolic disorders such as obesity, type 2 diabetes, and hypertension highlights the importance of dietary strategies for disease prevention and management, in which functional foods enriched with BAPs could play a significant role [1,2,3].

### 1.2. Why Pulses and Pseudocereals?

Different reviews have highlighted the numerous health benefits of constant consumption of vegetable proteins. The application of pulses and pseudocereals as raw materials for BAPs production is grounded in both nutritional value and strategic relevance. Pulses that contain 12–38% high-quality protein with a high level of essential amino acids (EAAs), particularly lysine represent a sustainable source of peptides with potential health-promoting effects [4,5,6,7]. Pseudocereals have garnered interest for their high-quality gluten-free protein content (up to 10–20%), balanced amino acid profiles, and unique functional properties that position them as promising alternatives to conventional cereals [8,9,10]. From a sustainability perspective, pulses and pseudocereals also contribute to environmentally friendly food systems: pulses improve soil fertility through nitrogen fixation and have among the lowest greenhouse gas emissions per gram of protein, while pseudocereals are resilient crops capable of thriving in marginal and climate-stressed environments [11]. In legumes, storage proteins, particularly globulins and albumins, as well as lectins and protease inhibitors, represent the primary reservoirs of BAPs. Similarly, in pseudocereals, 11S globulins and albumins play a central role as precursors of health-promoting peptides [10,12,13,14].

However, despite their advantages, these protein sources remain comparatively less studied than dairy, soy, or animal-derived proteins for peptide discovery. Critical gaps persist regarding standardized hydrolysis strategies, bioavailability, and *in vivo* validation of identified peptides, particularly from pseudocereals. By focusing on these underutilized crops, research not only diversifies the portfolio of BAP-producing raw materials but also aligns with global priorities of sustainability, dietary diversification, and chronic disease prevention. Thus, pulses and pseudocereals represent both an opportunity and a challenge-requiring deeper exploration to fully harness their potential in functional foods and nutraceutical applications.

### 1.3. Structural and Functional Relevance of Bioactive Peptides

Peptide sequence and length play a critical role in determining the bioactivity, stability, and structural characteristics of BAPs. According to literature, the relationship between peptide length and bioactivity is not simple. Instead, bioactivity is consistently observed within a specific, short length range, and the optimal length often depends on the specific biological function and mechanism of action. Indeed, most BAPs consist of 2 to 20 amino acids long with a molecular weight <3 kDa, and di- and tripeptides, for instance, often exhibit strong antihypertensive or antioxidant effects. Shorter BAPs can also easily reach targets compared to the longer one due to their less steric hindrance. Conversely, longer peptides (>20 residues) may develop secondary or tertiary domains that influence their bioactivity through specific conformational arrangements. Additionally, peptides with shorter lengths and lower molecular weights tend to be more resistant to gastrointestinal degradation, thereby maintaining functional activity *in vivo*. Overall, while shorter peptides generally display superior bioactivity, the specific amino acid sequence and structural context remain decisive factors for their functional potency. Many functional peptides have sequences rich in proline (P), leucine (L), arginine (R) and lysine (K). Health effects are linked to specific motifs, for example, the sequences VPP or IPP are linked to antihypertensive effects due to their angiotensin-converting enzyme (ACE) inhibition action, meanwhile sequences containing glutamine (Q)- or cysteine (C)-rich domains often exhibit antioxidant properties [3,10,13,15,16].

### 1.4. Aim and Scope of This Review

This review provides an integrated overview of recent advances in this field, beginning with the application of *in silico* tools for the prediction of peptide sequence bioactivities and structure–function relationships, which have accelerated peptide discovery and reduced experimental costs. It then examines bio-based processing approaches, particularly enzymatic hydrolysis and microbial fermentation, that enable the efficient release of BAPs from plant protein matrices through environmentally friendly and scalable technologies. Furthermore, the subject of bioactivity screening is addressed, with reference to the potential of plant-based BAPs in the regulation of some human metabolic pathways associated with various diseases, including type 2 diabetes, hypertension and obesity. Beyond the scientific and technological dimensions, this review explores the regulatory frameworks governing peptide-based food ingredients across the European Union, the United States, China, and Japan, underscoring the importance of harmonized standards for safety and health claims. Finally, production and commercialization hurdles are analyzed, highlighting the need for interdisciplinary strategies that couple computational design, bioprocess optimization, and regulatory innovation to unlock the full potential of plant-derived peptides in health-oriented food systems.

## 2. Bioinformatic Approaches for Bioactivity Prediction

**Figure 1:**
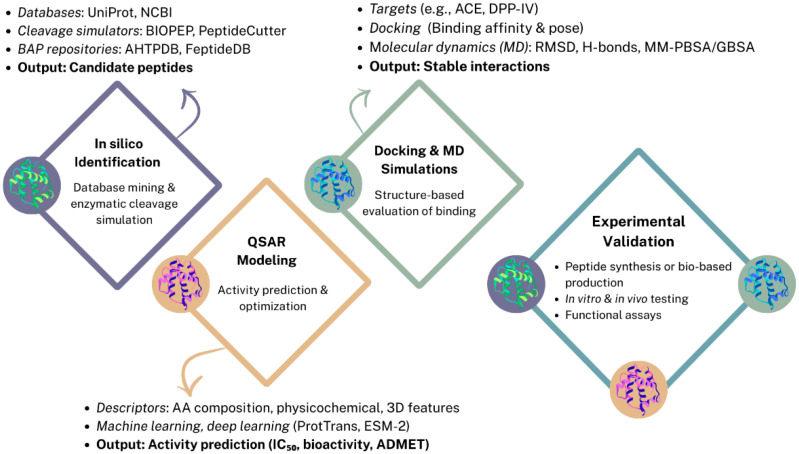
Bioinformatic approaches for BAPs prediction.

*In silico* prediction methods have emerged as powerful tools for the identification and characterization of BAPs derived from pulses and pseudocereals. These computational approaches enable the efficient screening of extensive protein databases and the prediction of peptide sequences with potential physiological benefits. By simulating enzymatic hydrolysis and assessing structural and physicochemical parameters, *in silico* tools facilitate the preliminary evaluation of peptides for specific bioactivities such as antihypertensive, antioxidant, antidiabetic, and anticancer effects prior to experimental validation (Figure 1). This strategy significantly accelerates the discovery pipeline, reducing the time and resources required for laboratory testing [10,17,18].

### 2.1. In Silico Identification of BAPs

The identification of BAPs pulses and pseudocereals has been greatly enhanced by bioinformatics tools that can simulate enzymatic hydrolysis and predict peptide sequences with potential functionality. Traditionally, peptide discovery was based on enzymatic hydrolysis and purification, involving labor-intensive experimental workflows, which limited throughput and scalability. *In silico* approaches enable rapid screening of food protein sequences using curated databases, enzymatic cleavage simulation platforms, and repositories of experimentally validated BAPs. Among the most frequently used tools, BIOPEP-UWM™ allows virtual hydrolysis by enzymes such as pepsin, trypsin, or Alcalase, and calculates indices such as the frequency of bioactive fragments (A_E_) and the potential biological activity (B_E_). PeptideCutter, Rapid Peptides Generator, and FeptideDB similarly predict enzymatic cleavage patterns, while AHPP and R-PeptideCutter simulate gastrointestinal digestion considering enzyme order and specificity. These resources, together with curated repositories such as AHTPDB and MLBP, facilitate the prediction of BAPs with antioxidant, antihypertensive, antidiabetic, or antimicrobial properties. Further insight into peptide–target interactions is provided by molecular docking and molecular dynamics (MD) simulations, which estimate binding affinities and reveal structural stability *in silico*. Integrated strategies combining proteolytic simulation, docking, and MD are increasingly used to optimize candidate selection before experimental validation [17,19,20,21].

### 2.2. Activity Prediction and Peptide Optimization via QSAR Modeling

Quantitative structure–activity relationship (QSAR) models offer a complementary layer of prediction by correlating peptide structural features with biological activity. These computational models use descriptors such as amino acid composition, physicochemical properties, or 3D conformational features to predict outcomes like inhibitory concentration (IC_50_), receptor binding potential, or binary classification of bioactivity. QSAR approaches can be regression-based, classification-based, or increasingly, deep learning–driven. Classical methods such as Multiple Linear Regression (MLR), Partial Least Squares Regression (PLSR), Support Vector Machine Regression (SVMR), and Random Forest Regression (RFR) are still applied for small datasets, whereas advanced Artificial Neural Networks (ANN) and Convolutional Neural Networks (CNN) are employed for large-scale modeling. Descriptor encoding remains central to QSAR performance. Local descriptors (e.g., FASGAI, z-scale indices) characterize amino acid–specific features, while global descriptors such as Comparative Molecular Field Analysis (CoMFA) and Comparative Molecular Similarity Indices Analysis (CoMSIA) account for 3D structural interactions. Recent advances include the use of protein language models (pLMs) such as ESM-2 and ProtTrans, which allow accurate feature extraction from variable-length peptides. Although dataset limitations remain a bottleneck, particularly the lack of high-quality negative samples and curated training data, QSAR models are now widely employed to screen large libraries of peptide candidates, prioritize sequences for synthesis, and reduce experimental costs. In BAP research, QSAR is especially valuable for predicting multi-bioactivity profiles and assessing properties such as allergenicity, organoleptic characteristics, absorption, distribution, metabolism, excretion, and toxicity (ADMET), paving the way for the rational design of functional peptides [17,18,21,22,23].

### 2.3. Structure-Based Approaches: Molecular Docking and Dynamics

While peptide identification and QSAR modeling offer sequence- and activity-level predictions, molecular docking and molecular dynamics (MD) simulations provide structure-based insights on how BAPs interact with their biological targets [20]. Docking simulations predict the preferred binding conformations and affinities of peptides within enzyme active sites or receptor pockets, commonly targeting ACE and dipeptidyl peptidase-IV (DPP-IV) due to their relevance in hypertension and diabetes management. Commonly employed programs include AutoDock Vina, AutoDockFR, GLIDE, Genetic Optimisation for Ligand Docking (GOLD), and HADDOCK, which utilize genetic algorithms or empirical scoring functions to rank peptide–receptor complexes. These methods enable virtual screening of multiple peptide candidates derived from *in silico* hydrolysis or mass spectrometry workflows. However, scoring functions in docking software are limited in accuracy and may not fully capture the flexibility of short peptides [17,21,24].

To overcome these limitations, MD simulations are increasingly used to refine docking results by modeling peptide–receptor complexes under physiological conditions over time. MD software such as GROMACS allows evaluation of complex stability, conformational changes, and key interactions at the atomic level. Techniques such as root-mean-square deviation (RMSD) analysis, hydrogen bond profiling, and binding free energy estimations Via molecular mechanics Poisson–Boltzmann surface area (MM-PBSA) or molecular mechanics generalized Born surface area (MM-GBSA) provide more reliable predictions of peptide efficacy. Structural visualization and validation are supported by PyMOL, Chimera, LigPlot+, and VMD, while 3D peptide models are generated using PEP-FOLD, PEPstrMOD, or AlphaFold [21].

Although computationally intensive, these approaches offer critical mechanistic insights that help bridge the gap between *in silico* predictions and biological function, guiding the rational design and validation of functional food-derived peptides [17].

### 2.4. In Silico Approaches for Pulses- and Pseudocereal-Derived BAPs: Case Studies

To exemplify the versatility and predictive power of *in silico* strategies in bioactive peptide discovery, four case studies highlight how computational hydrolysis, molecular docking, and ADMET modeling can efficiently identify peptides with promising antioxidant and enzyme-inhibitory properties.

In the study conducted by Lammi et al. (2016) [25] on soy and lupin, BIOPEP-based *in silico* prediction identified short peptide motifs (e.g., VA, VP, IA, GV, YV, FP, AE, PG, TF) with potential DPP-IV inhibitory properties. Peptides were modeled Via Monte Carlo simulations and docked against DPP-IV using PLANTS software, focusing on the Omarigliptin drug binding site (PDB ID: 4PNZ). The resulting complexes showed stable interactions within the catalytic region, supported by molecular dynamics simulations [25].

Arámburo-Gálvez et al. (2022) performed a study on enzymatic hydrolysis of chickpea legumin and provicilin sequences that was simulated using the BIOPEP-UWM™ database, identifying ACE-inhibitory peptides later validated through molecular docking (PDB ID: 1O86) and ADMET analysis, made using ADMETLab 2.0, confirming good oral bioavailability and pharmacokinetic properties [26]. Similarly, *in silico* pipelines applied to chickpea proteins in the study of Mora-Melgem et al. (2023) [27] have revealed peptides with strong DPP-IV inhibitory potential. Protein sequences of legumin and provicilin were hydrolyzed virtually using BIOPEP-UWM™ (Olsztyn-Kortowo, Poland) with enzymes such as Alcalase, papain, bromelain, ficin, pepsin, trypsin, and chymotrypsin, including simulated gastrointestinal digestion. The resulting fragments were docked against DPP-IV (PDB ID: 4PNZ), validated by re-docking the crystallographic inhibitor Omarigliptin drug with a low RMSD (0.972 Å). The best peptide–enzyme complexes were visualized in Discovery Studio, and pharmacokinetic analyses Via ADMETlab 2.0 indicated good absorption, moderate distribution, and low toxicity. These findings underscore the predictive strength of *in silico* hydrolysis and docking for identifying functionally relevant chickpea peptides prior to experimental validation [27].

Similarly, peptidomic-based *in silico* pipelines applied on *Pisum sativum* hydrolysates combined Nano-LC-MS/MS profiling and AutoDock Vina docking to identify over 500 peptides (94% below 1.6 kDa) with strong DPP-IV binding affinities. Peptides containing proline or alanine at the second N-terminal position, such as IPYWTY and LPNYN, showed the highest predicted inhibitory potential against DPP-IV (PDB ID: 1WCY) [28].

Expanding this approach to pseudocereals, in a recent *in silico*-guided study on quinoa 11S-globulin hydrolyzed by stem bromelain exemplifies how bioinformatics can streamline peptide discovery. Computational hydrolysis generated 109 fragments, of which 14 peptides were shortlisted through molecular docking and dynamics simulations targeting ACE-I, DPP-IV, α-glucosidase, and lipoxygenase. Among these, NIYQIS and QDQHQKIR exhibited the strongest binding affinities, stable hydrogen-bonding interactions, and favorable ADMET properties, including good blood–brain barrier permeability and low predicted toxicity. Subsequent *in vitro* validation confirmed NIYQIS as the most promising multifunctional peptide, combining potent ACE inhibition (53%) and DPP-IV (16.36%) inhibition with strong antioxidant capacity (0.75 µM TE/µM peptide). QDQHQKIR also demonstrated notable α-amylase inhibition (18.43%) and Cu^2+^ chelation (40.4%), underscoring its relevance against metabolic and oxidative stress disorders [29].

Together, these studies illustrate how integrating *in silico* hydrolysis, peptidomics, and molecular docking with pharmacokinetic modeling enables the efficient identification of BAP candidates from legumes and pseudocereals. Such computational pipelines not only accelerate discovery but also support the rational design of peptide-based functional ingredients targeting hypertension, diabetes, and oxidative stress paving the way for their translation into next-generation nutraceuticals. Looking ahead, the integration of *in silico* predictive tools with fermentation-based strategies represents a promising yet largely unexplored frontier. Computational modeling could be employed to predict microbial proteolytic patterns, in order to optimize fermentation parameters, and guide strain selection toward the targeted release of specific BAPs. However, translating these predictions into experimentally validated outcomes remains a significant challenge, underscoring the need for deeper interdisciplinary research that bridges bioinformatics, microbiology, and food biotechnology.

## 3. Bio-Based Approaches for the Production of BAPs

**Figure 2:**
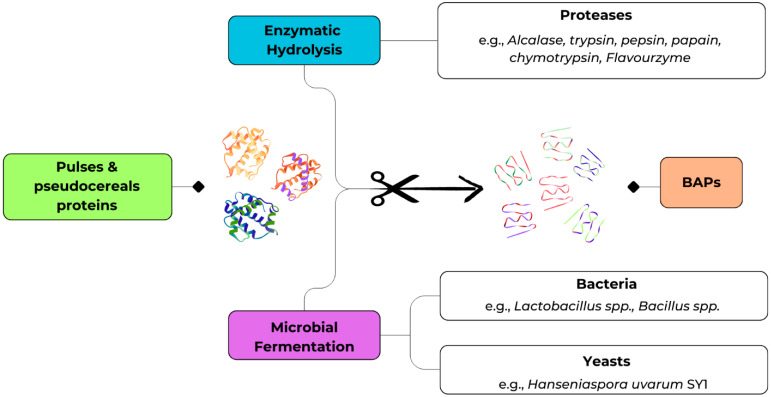
Bio-based strategies for the production of BAPs.

The production of BAPs from pulses and pseudocereals has been predominantly achieved through two bio-based strategies, namely enzymatic hydrolysis and microbial fermentation as shown in Figure 2 [30]. Enzymatic hydrolysis relies on the use of exogenous proteases to release specific peptide sequences under controlled conditions, whereas microbial fermentation exploits the proteolytic machinery of selected microorganisms to liberate peptides during growth.

These approaches are not mutually exclusive and, in fact, differ substantially in terms of efficiency, scalability, and cost. Enzymatic hydrolysis provides precise and reproducible control over proteolysis but is often limited by enzyme cost and the need for downstream processing. Conversely, fermentation is slower and less predictable, yet it is economically attractive, sustainable, and capable of improving both nutritional and sensorial attributes. The following sections synthesize the most recent evidence on enzymatic and fermentation-based production of BAPs from pulses and pseudocereals, with emphasis on bioactivity, structural characterization, and technological relevance.

### 3.1. Enzymatic Treatments

Enzymatic hydrolysis has been the method of choice for most mechanistic studies, as it enables the targeted release of short peptides under defined physicochemical conditions. Proteases such as Alcalase, trypsin, pepsin, papain, chymotrypsin, and Flavourzyme are widely employed, and the degree of hydrolysis can be modulated, mainly setting the time of hydrolysis and combining different enzymes, to generate peptides of desired molecular size and bioactivity [27,31]. A consistent finding across legume and pseudocereal proteins is that low-molecular-weight peptides (<3 kDa) are enriched in hydrophobic, aromatic, or proline residues, features strongly associated with ACE and DPP-IV inhibition. As illustrated in Table 1, there is a wide range of applications of enzymatic hydrolysis on different pulses and pseudocereal matrices, with the subsequent production and bioactivity outcomes. This paragraph will provide a description of these applications.

Production of BAPs from chickpea has been highlighted by Mora-Melgem et al. (2023) [27], who reported that hydrolysis with papain and trypsin generated 92 peptides with predicted DPP-IV inhibitory capacity, of which a substantial proportion also exhibited ACE inhibition. The dipeptide His–Phe was singled out as the most potent inhibitor, consistent with its ability to interact with key catalytic residues of DPP-IV [27]. Moreover Quintero-Soto et al. (2021) [32] showed the antidiabetic properties of albumin and globulin fractions of chickpea subjected to Alcalase hydrolysis and chromatographic purification. They reported the yield of short sequences (FEI, FIE, and FEL) that inhibited DPP-IV with an IC_50_ of 0.0042 mg/mL, while FGKG preferentially targeted carbohydrate-digesting enzymes, reducing α-glucosidase and α-amylase activities by 56% and 54%, respectively [32].

Lentil proteins subjected to simulated gastrointestinal digestion released peptides such as KLRT, TLHGMV, and VNRLM, which displayed strong ACE-inhibitory activity (IC_50_ = 0.0197 mg/mL), confirming the value of gastrointestinal stable lentil peptides as antihypertensive agents [33]. In addition, Garcia-Mora et al. (2015) [34] reported that lentil proteins subjected to enzymatic hydrolysis with different proteases, in combination with high-pressure treatment, generated peptides with marked bioactivities. Among the tested conditions, Savinase-assisted hydrolysis at 300 MPa produced the most potent effects, yielding the highest ACE-inhibitory activity (69.5%) together with a strong antioxidant capacity (403.86 μmol TE/g). These findings indicate that pressure-assisted enzymatic hydrolysis can significantly enhance the release of multifunctional peptides from lentil proteins [34].

Bollati et al. (2022) [35], showed that commercial pea protein contains peptides with dual ACE- and DPP-IV-inhibitory potential. In fact, the pea protein hydrolysate inhibited DPP-IV and ACE *in vitro* with IC_50_ values of 1.33 and 0.61 mg/mL, respectively, while its <3 kDa fraction displayed even stronger activity (IC_50_ = 1.0 mg/mL for DPP-IV and 0.43 mg/mL for ACE). Cell-based experiments confirmed these findings, as pea protein hydrolysate reduced the activity of both enzymes in Caco-2 cells in a dose-dependent manner without cytotoxic effects [35]. Ásledóttir et al. (2023) [36] evaluated the gastrointestinal stability and bioactivity of pea and faba bean proteins digested with standardized human gastric and duodenal juices (INFOGEST). Peptidomic analysis predicted the release of 275 BAPs from pea and 268 from faba bean. From these, selected sequences such as DKPWWPK and NEPWWPK (pea), together with QQGPPPPPPPISL and ATPPPPPPPMSL (faba bean), were further characterized in cell assays. All tested peptides significantly reduced IL-8 secretion by up to ~40%, highlighting their immunomodulatory potential and suggesting that both pea and faba bean proteins represent promising dietary sources of immune-active peptides [36].

Quinoa proteins also yield multifunctional peptides. Hydrolysis with chymotrypsin produced peptides with inhibitory effects on cholesterol esterase and pancreatic lipase, with sequences such as QHPHGLGALCAAPPST and HVQGHPALPGVPAHW identified as active binders. These results suggest that quinoa-derived peptides may exert antihyperlipidemic effects by targeting lipid metabolism enzymes [31]. Additional studies demonstrated that simulated gastrointestinal digestion of quinoa proteins produced fractions with both ACE-inhibitory and antioxidant activity, underscoring the versatility of quinoa peptides [37]. The antidiabetic activity of quinoa proteins has been investigated by Abbasi et al. (2022) [38], who reported that hydrolysates obtained through sequential treatment with Alcalase and trypsin exhibited inhibitory effects on carbohydrate-digesting enzymes. In particular, the fraction with molecular weight ≥3 kDa generated after 0.5 h of hydrolysis displayed the strongest activity, achieving 44.8% inhibition of α-glucosidase [38].

Ayala-Niño et al. (2019) demonstrated that sequential hydrolysis with Alcalase and Flavourzyme of amaranth yielded fractions displaying not only ACE-inhibitory activity (IC_50_ = 0.134–0.808 mg/mL) but also thrombin inhibition and substantial antioxidant activity, thereby illustrating the multifunctionality of amaranth peptides in cardiovascular health [39].

Soy and lupin proteins are among the most extensively investigated, particularly in relation to metabolic health. Lammi et al. (2019) showed that soy protein hydrolysates below 3 kDa inhibited HMG-CoA reductase, upregulated LDL receptor expression, and inhibited DPP-IV activity in Caco-2 cells, with peptides such as IAVPTGVA, IAVPGEVA, and LPYP identified as active contributors [40]. Earlier work by Lammi et al. (2016) [25] reported that IAVPTGVA (Soy1) and LTFPGSAED (Lup1) acted as potent DPP-IV inhibitors, with molecular docking analyses confirming their interaction with catalytic sites. Collectively, these results highlight the relevance of soy and lupin peptides in the modulation of glycemic control and lipid metabolism [25]. Moreover, the bioactivity of soybean protein hydrolysate has been investigated by Bollati et al. (2022) [35], who demonstrated that peptidomic analysis revealed the presence of sequences with both ACE- and DPP-IV-inhibitory motifs. *In vitro* assays confirmed a strong multifunctional profile, with soybean hydrolysate inhibiting DPP-IV (IC_50_ = 1.15 mg/mL) and ACE (IC_50_ = 0.33 mg/mL). The <3 kDa peptide fraction further enhanced these effects (IC_50_ = 0.82 and 0.40 mg/mL, respectively), highlighting the role of short peptides in bioactivity. Importantly, soybean hydrolysate also suppressed DPP-IV and ACE activity in Caco-2 cells without impairing cell viability, confirming its potential as a multifunctional anti-diabetic and antihypertensive ingredient [35]. Soy hydrolysate was also reported by Boschin et al., (2014) [41] who showed that peptic hydrolysis of soybean proteins (pepsin digestion, 18 h, E:S = 1:100; <3 kDa fraction) generated peptide mixtures with strong ACE-inhibitory activity. The hydrolysate inhibited ACE in a dose-dependent manner, with an IC_50_ of 0.224 mg/mL and reaching 88% inhibition at the highest concentration tested (0.983 mg/mL). Under the same experimental conditions, pea proteins showed moderate ACE-inhibitory activity compared with soybean. The hydrolysate achieved an IC_50_ of 0.595 mg/mL and a maximum inhibition of 71% at the highest concentration tested (0.861 mg/mL) [41]. Coscueta et al. (2019) [42] showed that soybean proteins hydrolyzed with Corolase PP (1% E:S, 4 h, 50 °C) released BAPs with dual functionality. The resulting hydrolysates exhibited enhanced antioxidant activity (3.9 ± 0.1 μmol TE/mg) and a strong ACE-inhibitory effect (IC_50_ = 0.052 mg/mL), confirming their potential as multifunctional ingredients for managing oxidative stress and hypertension [42].

The efficient production of BAPs from legumes and pseudocereals relies on optimized E:S ratios, enzyme specificity, and reaction conditions. Most studies report effective hydrolysis with E:S ratios from 2 to 10% (*w*/*w*), maintaining enzyme-specific pH (7–9) and moderate temperatures (50–56 °C) for 2–6 h. Alcalase, Flavourzyme, and dual-enzyme blends are frequently used, as their broad specificity promotes the release of diverse BAPs. Excessive enzyme addition or prolonged hydrolysis often provides no further benefit, while mild pre-treatments, such as heating, can enhance hydrolysis reaction and peptide yield. Overall, conditions tailored to enzyme optima and balanced E:S ratios ensure high bioactivity and hydrolysis efficiency, supporting their application in functional food and nutraceutical development. Although enzymatic hydrolysis enables reproducible peptide production and in-depth characterization, its limitations include enzyme costs, potential formation of bitter hydrophobic peptides, and the need for inactivation and purification steps. Nonetheless, technological advances such as ultrasound- or high-pressure–assisted hydrolysis are increasingly being employed to improve peptide yield and bioactivity [43].

**Table 1:** Bioactive peptides (BAPs) obtained through enzymatic hydrolysis of pulse and pseudocereal proteins, with details on production methods and reported bioactivities.

Source	BAPs Production		Bioactivity				Ref
	Treatments	Outcome	Type	Dosage	Control	Outcome	
Adzuki bean	Flavourzyme	Adzuki F2 fraction	Antimicrobial	2 mg/mL	Gentamicin, chloramphenicol	*S. typhimurium* inhibition (76%)	[44]
Amaranth	Sequential Alcalase + Flavourzyme hydrolysis	Bioactive peptides identified in fraction “45” (e.g., HVQLGHY, SQIDTGS, NWACTL)	Antihypertensive/Antithrombotic/Antioxidant	10 mg/mL amaranth protein hydrolysate	Hippuric acid; thrombin; unhydrolyzed amaranth proteins	Multifunctional: ACE inhibition (IC_50_ = 0.134–0.808 mg/mL), thrombin inhibition (IC_50_ = 0.155–0.167 mg/mL), ABTS antioxidant SC_50_ = 0.992–6.931 mg/mL;	[39]
Chickpea	*In silico* enzymatic hydrolysis (papain and trypsin)	Prediction of ninety-two peptides with potential DPP-IV and ACE inhibition activity	Antidiabeitc/Antihypertensive	NA	Omarigliptin (PDB ID: 4PNZ)	His–Phe identified as most potent; *in silico* predicted DPP-IV and ACE-inhibitory	[27]
Chickpea	Alcalase hydrolysis of albumin and globulin; chromatographic purification	Bioactive sequences identified (e.g., FEI, FIE, FEL and FGKG)	Antioxidant/Antidiabetic	1 mg/mL for hydrolysate; 0.2 mg/mL for peptide fraction; 0.1 mg/mL for α-amylase inhibition assay	Acarbose (1 mmol/L); reaction buffer	High radical scavenging (ABTS and DPPH); FEI, FIE FEL had DPP-IV inhibition (IC_50_ = 0.00420 mg/mL) while FGKG showed α-glucosidase and α-amylase inhibition (56% and 54%)	[32]
Cowpea seed	Enzymatic hydrolysis on the protein isolate with Alcalase, 1:200 (E:S), 4 h, 55 °C, pH 7.8; SEC purification	Cowpea seed protein hydrolysate (<1 kDa)	Antimicrobial	0.025–0.150 mg/mL	Ciprofloxacin	Broad-spectrum antimicrobial, membrane disruption confirmed	[45]
Faba bean	Simulated digestion with human gastric & duodenal juices (INFOGEST)	268 BAPs predicted	Anti-inflammatory	0.1–1000 µM	IL-1β with positive, negative (Fresh serum-free growth medium), and IL-1Rα controls	Selected peptides (e.g., QQGPPPPPPPISL, ATPPPPPPPPMSL) reduced IL-8 up to ~40%, indicating immunomodulatory activity	[36]
Black Jampa bean	Hydrolysis on protein isolate (5%, *w*/*v*) using pepsin (90 min, pH 2.5) followed by pancreatin (120 min, pH 7.5) at 1:20 *w*/*w*, E:S	Bean protein hydrolysate fractions (phaseolin-rich)	Antioxidant	100 μg hydrolysates or 50 μg peptide fractions	Blank	0.7–1.0 kDa peptides had highest Cu^2+^ chelation and moderate Fe^2+^ binding	[46]
Lentil	*In vitro* simulated GI digestion of lentil flour; ion-exchange and gel filtration fractionation	Fraction 5 contained peptides KLRT, TLHGMV, VNRLM	Antihypertensive	50 µL of the peptide sample	Hippuryl-His-Leu solutions (1.0, 2.0, 3.0, 4.0 and 10.0 mm)	ACE inhibition, IC_50_ = 0.0197 mg/mL	[33]
Lentil	Protamex, Savinase, Corolase and Alcalase hydrolysis assisted with high-pressure	The process increased the concentration of peptides under 3 kDa, particularly with pressurization at 300 MPa by all enzymes tested	Antihypertensive/Antioxidant	0.5 mg/mL (for ACE inhibition assay)	Non-hydrolyzed lentil proteins	Highest ACE-inhibitory (69.46%) and antioxidant activity (403.86 μmol TE/g) through Savinase treatment at 300 MPa	[34]
Lupin	Enzymatic *in silico* prediction/ peptide synthesis	LTFPGSAED (Lup1), IC_50_ = 228 µM	Antidiabetic	Concentrations ranging from 0.01 to 1 mM	Sitagliptin (0.0001 mM)	DPP-IV inhibitory; docking confirmed binding; other lupin peptides inactive	[25]
Lupin	Lupin protein isolate (10% *w*/*v*) hydrolyzed with Alcalase 2.4 L (15 min at pH 8, 50 °C, and E:S = 0.3 AU/g protein)	Lupin protein hydrolysate	Antidiabetic	100 mg/kg (mice), 1 g/day 28 days (human)	Placebo	Inhibited DPP-IV, improved glucose control in mice and humans	[47]
Lupin	Sequentially digested with pancreatin (pH 7.5) and pepsin (pH 2.0), in a ratio of 1:20 *w*/*w* (E:S) at 37 °C for 1 h	Andean lupin γ-conglutin hydrolysate	Antidiabetic	5 mg/mL	Untreated cells; metformin (1 mM) and insulin (100 nM)	Inhibited DPP-IV, ↑GLUT4 translocation, ↓gluconeogenesis	[48]
Lupin	Alcalase hydrolysis for 15 min at pH 8, 50 °C, and E:S = 0.3 AU/g protein	Lupin protein hydrolysate	Anti-inflammatory	0.1–0.5 mg/mL	LPS-treated co-culture, unstimulated cells	Blunted TNF-α, IL-1β, IL-6 upregulation, IL-1β below baseline	[49]
Lupin	Alcalase hydrolysis on lupin protein isolate for 15 min	Lupin protein hydrolysate	Anti-inflammatory	100 mg/kg oral prophylactic	EAE mice untreated	Reduced severity, better neurologic function	[50]
Lupin	Alcalase 2.4 L (E:S = 0.3 AU/g protein); pH 8.0 at 50 °C for 15 min	Lupin protein hydrolysate	Prebiotic	100 mg/kg/day	HFD mice untreated	Reduced obesity, improved metabolism, restored *Akkermansia* abundance	[51]
Mung bean	Flavourzyme	Mung bean F4 fraction	Antimicrobial	2 mg/mL	Gentamicin, chloramphenicol	*S. aureus* inhibition (71%)	[44]
Pea	Industrial hydrolysis, ultrafiltration	<3 kDa peptide fraction	Antidiabetic/Antihypertensive	DPP-IV inhibitory assay: *in vitro =* 0.01 to 2.0 mg/mL; cellular assay = 1.0, 2.5, and 5.0 mg/mL; ACE inhibitory activity: *in vitro* = 0.08, 0.17, 0.35, 0.7, and 1.0 mg/mL; cellular assay = 0.1 to 5.0 mg/mL	Control (C) sample; growth medium and H_2_O	Inhibited DPP-IV (IC_50_ = 1.33 mg/mL; <3 kDa fraction IC_50_ = 1.0 mg/mL) and ACE (IC_50_ = 0.61 mg/mL; <3 kDa fraction IC_50_ = 0.43 mg/mL); active in Caco-2 cells	[35]
Pea	Simulated digestion with human gastric & duodenal juices (INFOGEST)	275 BAPs predicted	Anti-inflammatory	0.0001–1 mM	IL-1β with positive, negative (Fresh serum-free growth medium), and IL-1Rα controls	Selected peptides (e.g., DKPWWPK, NEPWWPK) reduced IL-8 up to ~40%, indicating immunomodulatory activity	[36]
Pea	Pepsin hydrolysis of total protein extract (18 h, E:S = 1:100), ultrafiltration <3 kDa	Peptide mixtures with ACE-inhibitory activity	Antihypertensive	0.861 mg/mL	Inhibitor Blank (AIB): enzyme but no inhibitor; Reaction Blank (ARB): highest inhibitor concentration but no enzyme.	ACE inhibition: IC_50_ = 0.595 mg/mL; maximum inhibition 71% at the highest concentration tested (0.861 mg/mL)	[41]
Quinoa	Papain digestion of quinoa bran globulin powder	Quinoa bran globulin hydrolysate (SAPPP fraction)	Antihypertensive	IC_50_ = 0.915 mM	NA	Stable ACE inhibition after digestion, pH fluctuations (2.0–10.0), pasteurization conditions, addition of ions	[52]
Quinoa	Chymotrypsin hydrolysis (QPI, 2 h)	Peptides identified (e.g., QHPHGLGALCAAPPST)	Anti-hypercholesterolemic	25–50 μL of sample	*p*-nitrophenyl butyrate	Highest inhibition of cholesterol esterase (IC_50_ = 0.51 mg/mL) and pancreatic lipase (IC_50_ = 0.78 mg/mL	[31]
Quinoa	Simulated GI digestion of protein isolates	Peptide fractions < 5 kDa and > 5 kDa	Antioxidant	NA	Trolox (0.2–1.6 nmol);	Peptides with antioxidant and colon cancer cell viability inhibitory activity identified; hydrolysates showed strong radical-scavenging activity	[37]
Quinoa	Alcalase and trypsin hydrolysis; MW cut-off = 3, 10 kDa–	Different molecular weight peptide fractions	Antidiabetic	NA	pNPG substrates	Highest α-glucosidase inhibition (44.8%) obtained with 0.5 h hydrolysis time and 3 kDa ≥ MW.	[38]
Quinoa	High pressure-assisted enzymatic hydrolysis with 0.14 AU of Alcalase/ g protein (1:20 *w*/*v* E:S)	Quinoa protein hydrolysate (<3 kDa)	Antihypertensive	0.1–0.5 mg/mL	Blank, non-hydrolyzed proteins, conventional hydrolysis	High-pressure hydrolysis improved ACE inhibition	[53]
Quinoa	Simulated gastro-intestinal digestion, only pepsin and pancreatin	Quinoa albumin peptides (lunasin-rich)	Anti-inflammatory	1 mg/mL	IL-1β-treated cells	Up to 74% reduction in NF-κB activity. *In vitro* digestion enhanced the effect	[14]
Red quinoa	Hydrolysis with Alcalase 2.4 LFG, 2 h.	Red quinoa protein hydrolysate	Antioxidant/ Antihypertensive	1000 mg/kg/day (8 weeks)	Hypertensive rats untreated (water) or with vitamin C	Increased glutathione, decreased MDA, improved systemic antioxidant status, reduction in systolic blood pressure	[54]
Soy	Hydrolysis with pepsin and trypsin; ultrafiltration (<3 kDa)	Peptic (P) and tryptic (T) soybean hydrolysates	Antidiabetic	Range of 0.5–2.5 mg/mL	NA	HMG-CoA reductase inhibition (up to −77%), ↑LDL receptor expression and LDL uptake, DPP-IV inhibition (up to 43% in Caco-2); peptides characterized by LC-MS/MS	[40]
Soy	Enzymatic *in silico* prediction/peptide synthesis	Glycinin hydrolysis (Soy1 = IAVPTGVA), IC_50_ = 106 µM	Antidiabetic	Concentrations ranging from 10 to 1000 μM	Sitagliptin (0.1 μM)	DPP-IV inhibitory peptide; docking confirmed binding	[25]
Soy	Industrial hydrolysis, ultrafiltration	<3 kDa peptide fraction	Antidiabetic/Antihypertensive	DPP-IV inhibition: 0.01–2.0 mg/mL (*in vitro*), 1.0–5.0 mg/mL (cells); ACE inhibition: 0.08–1.0 mg/mL (*in vitro*), 0.1–5.0 mg/mL (cells).	Control (C) sample; growth medium and H_2_O	Inhibited DPP-IV (IC_50_ = 1.15 mg/mL; <3 kDa fraction IC_50_ = 0.82 mg/mL) and ACE (IC_50_ = 0.33 mg/mL; <3 kDa fraction IC_50_ = 0.40 mg/mL); active in Caco-2 cells	[35]
Soy	Corolase PP hydrolysis (1% E:S, 4 h, 50 °C)	Soy protein hydrolysate as biofunctional ingredient	Antihypertensive/Antioxidant	40 μL of sample	Trolox; ACE + Abz-Gly-Phe(NO2)-Pro	Improved antioxidant activity (3.9 ± 0.1 μmol TE/mg) and ACE inhibitory peptides (IC_50_ = 0.052 mg/mL)	[42]
Soy	Pepsin hydrolysis of total protein extract (18 h, E:S = 1:100), ultrafiltration <3 kDa	Peptide mixtures with ACE-inhibitory activity	Antihypertensive	0.983 mg/mL	Inhibitor Blank (AIB): enzyme but no inhibitor; Reaction Blank (ARB): highest inhibitor concentration but no enzyme.	ACE inhibition: IC_50_ = 0.224 mg/mL; maximum inhibition 88% at the highest concentration tested (0.983 mg/mL)	[41]
Soy	Simulation of Gastrointestinal Digestion	Germinated soybean peptides (5–10 kDa)	Antidiabetic	IC_50_ 0.91 mg/mL	Diprotin A-	DPP-IV inhibition, active sequences from β-conglycinin, glycinin, P34	[55]
Soy	Proteinase PROTIN SD-NY10 (EC 3.4.24.28), 0.05% *w*/*w*, 50–55 °C for 16 h	Soymilk hydrolysate tetrapeptide	Antihypertensive	80 μg/kg/day (3 weeks)	Untreated SHR	Lowered BP, strong ACE inhibition	[56]

NA = information not available.

### 3.2. Production of BAPs Through Fermentation

Fermentation represents a complementary bioprocess that exploits the proteolytic capacity of microorganisms, particularly lactic acid bacteria, to release BAPs. In addition to peptide liberation, fermentation may confer benefits such as improved flavor, reduced antinutritional factors content, and the generation of additional bioactive metabolites. This makes fermentation highly attractive for the development of functional foods, even if it is generally less predictable than enzymatic hydrolysis [30,57].

Different strategies are used to produce BAPs, and the results depend on the type of microorganism and the fermentation process required. Lactic acid bacteria (LAB), including *Lactiplantibacillus plantarum*, *Lacticaseibacillus rhamnosus*, and *Pediococcus acidilactici*, along with *Bacillus* spp., are commonly employed due to their strong protease activity and safety profile, while certain yeasts such as *Hanseniaspora uvarum* also show promising peptide-releasing potential. Techniques such as solid-state and liquid-state fermentation are applied, optimizing parameters such as moisture, inoculum size, temperature, and time according to the starting raw material. Pretreatments such as germination or ultrasonic treatment are also important for enhancing the availability and accessibility of proteins, thereby improving BAP yields [58,59].

As shown in Table 2, microbial fermentation has been explored in different pulse and pseudocereal matrices, resulting in diverse peptide production and bioactivities. In the following, we describe these applications more closely.

Chickpea proteins have been widely investigated as substrates for fermentation-driven bioactivity. Li and Wang (2021) showed that solid-state fermentation with *Bacillus subtilis* lwo promoted extensive proteolysis, generating low-molecular-weight peptides (<10 kDa, up to 25.8 mg/g after 12 h) and enhancing antioxidant capacity in a time-dependent manner [60]. Expanding on these findings, Chiacchio et al. (2025) demonstrated that lactic acid fermentation of chickpea puree with diverse LAB strains markedly improved the nutritional profile of the resulting flours, with increases in polyphenolic content and the release of BAPs including predicted DPP-IV and ACE inhibitors [61]. More recently, Xu et al. (2025) optimized fermentation with selenium-enriched *Bacillus natto*, achieving an ACE-inhibition rate of ~80.7% under specific conditions (2% inoculum, 19:1 liquid-to-solid ratio, 40 °C), highlighting the potential of Se-enrichment to combine nutritional fortification with antihypertensive functionality [62]. Collectively, these studies underscore the versatility of fermentation approaches to unlock antioxidant, antidiabetic, and antihypertensive peptides from chickpea proteins.

Studies on faba bean demonstrate the strong potential of fermentation. Jakubczyk et al. (2017) [63] fermented faba bean flour with *Lactiplantibacillus plantarum* 299 v at 30 °C for three days and identified multiple peptides in the <3 kDa fraction, which displayed potent ACE inhibition (IC_50_ = 0.05 mg/mL). These sequences contained di- and tri-peptide motifs (e.g., GL, DA, MY) previously associated with antihypertensive activity. In peas, the same group showed that seven-day fermentation with *L. plantarum* 299 v had limited effects on the intact protein, but subsequent gastrointestinal digestion released peptides that improved ACE-inhibitory activity (IC_50_ = 0.19 mg/mL versus 0.37 mg/mL in the control), with KEDDEEEEQGEEE identified as an active sequence [33,63,64].

Tonini et al. (2024) [65] explored the fermentation of red-lentil protein isolates with multiple LAB and yeasts, identifying strain-dependent differences in peptide release. The yeast *Hanseniaspora uvarum* SY1 produced the richest profile of BAPs, particularly those with antioxidant and ACE-inhibitory activities, underscoring the potential of non-conventional microbial starters for generating health-promoting peptides from lentils [65].

In lupin, Ayyash et al. (2019) reported that solid-state fermentation with *L. plantarum* K779 significantly improved both antioxidant and ACE-inhibitory activities compared to the unfermented controls, likely reflecting synergistic effects of liberated peptides and phytochemicals [66].

Fermentation also enhances the bioactivity of pseudocereals. Cruz-Casas et al. (2023) [67] investigated the impact of fermentation with various lactic acid bacteria and *Bacillus* spp. on amaranth proteins, obtaining hydrolysates with bioactive properties. The extracts displayed strong antioxidant activity (9.18 μM TE/L), notable ACE-inhibitory effects (80.65%), and antimicrobial activity against foodborne pathogens, underscoring amaranth as a versatile pseudocereal for multifunctional peptide production through fermentation [67]. Moreover, Rizzello et al. (2012) [68] demonstrated that amaranth fermentation with *Lactobacillus* spp. induced the production of lunasin, a BAP with documented anticancer and cholesterol-lowering activity [68].

Solid-state fermentation with *L. plantarum* K779 on quinoa, applied in the study of Ayyash et al. (2019), showed significant improvement in ACE-inhibitory activities when compared to the unfermented control [66]. Li et al. (2022) [69] reported that fermentation of quinoa flour with *Lactobacillus paracasei* CICC 20241 led to the release and identification of several antihypertensive peptides. Among them, NIFRPFAPEL and AALEAPRILNL were characterized as potent ACE inhibitors, with IC_50_ values of 49.02 µM and 79.72 µM, respectively. These findings confirm that microbial fermentation can unlock quinoa-derived peptides with significant potential for blood pressure regulation [69]. Buckwheat fermentation has also been optimized: Wang and Ma (2024) reported that solid-state fermentation with *L. plantarum* under optimized conditions increased peptide content to 22.2 mg/mL, outperforming enzymatic methods in both yield and sensory improvement [59].

The efficiency of BAP production through microbial fermentation depends on the careful selection of microbial strains, substrate type, and precise control of fermentation parameters. LAB strains such as *Lactiplantibacillus plantarum* and *Levilactobacillus brevis*, together with yeasts like *Hanseniaspora uvarum*, are especially effective owing to their robust proteolytic systems, adaptability to plant substrates, and generally recognized as safe (GRAS). *Bacillus subtilis* is another promising candidate, particularly in solid-state fermentation, due to its strong protease activity. Optimal conditions typically fall within moderate ranges: temperatures of 22–37 °C (with 30 °C often cited for maximizing peptide yield), neutral to slightly acidic pH values (6–7), and fermentation times that vary with the desired activity.

Shorter durations of *Lactobacillus plantarum* fermentation on beans, such as 3 h at 22 °C, have been shown to favor the production of α-amylase-inhibitory peptides. Conversely, longer fermentations, up to 72 h at 30 °C, have been observed to enhance ACE-inhibitory and antioxidant activities [70]. For SSF a moisture content of ~60% is considered optimal, particularly for pseudocereals such as buckwheat, and the inoculum size also plays an important role, with higher ratios (~12%) enhancing peptide release [59].

In summary, microbial fermentation represents a versatile and sustainable strategy for generating bioactive peptides from legumes and pseudocereals. The choice of microorganism, together with precise control of temperature, pH, moisture, and fermentation time, determines the peptide profile and resulting bioactivities. While longer fermentations often enhance peptide diversity and potency, they also carry the risk of over-hydrolysis. Despite limitations such as extended processing times, batch-to-batch variability, and the need for strict microbial management, fermentation remains a cost-effective, consumer-acceptable approach that not only boosts peptide production but also improves nutritional quality and functional value, making it a promising tool for the development of health-oriented foods.

**Table 2:** Bioactive peptides (BAPs) obtained through microbial fermentation of pulse and pseudocereal proteins, with details on production methods and reported bioactivities.

Source	BAPs Production		Bioactivity				Ref
	Treatments	Outcome	Type	Dosage	Control	Outcome	
Amaranth	Fermentation with various lactic acid bacteria and *Bacillus* spp.	Multi potential dought	Antihypertensive/Antimicrobial	5–10 µL of sample for antioxidant and ACE-inhibitory assays; 50 µL for antimicrobial activity assay	Control time 0 min (unfermented amaranth dough) and 24 min (spontaneously fermented dough)	antioxidant (9.18 μM TE/L), ACE-inhibitory (80.65%) and antimicrobial activities against pathogens	[67]
Amaranth seed protein hydrolysates	Fermentation with *Enterococcus* faecium vs. enzymatic hydrolysis	Protein hydrolysates contain novel peptides with antihypertensive activity	Antihypertensive	0.00625 mg/mL	Blank	*Enterococcus faecium* hydrolysate strongest (79% inhibition)	[71]
Buckwheat	Solid-state fermentation with *L. plantarum* (12.87% inoculum, 60% moisture, 31.4 °C, 6 d)	Peptide content 22.18 mg/mL under optimal conditions; fermentation produced high peptide levels (and better flavor)	NA	NA	NA	NA	[59]
Chickpea	Fermentation of 20% chickpea puree with 14 LAB strains; 48 h; flours obtained by freeze-drying	Flour with enhanced bioactive peptides	Antidiabetic/Antihypertensive	200 µL of the sample extract	Trolox	Higher polyphenolic content; BAPs incl. DPP-IV/ACE-inhibitor candidates	[61]
Chickpea	Fermentation with selenium-enriched *Bacillus natto*	Under optimized condition (2% inoculum, 19:1 liquid-to-solid ratio (mL/g), and 40 °C)	Antihypertensive	40 μL	HEPES (80 mmol/L)	ACE-inhibition rate ~80.7%	[62]
Chickpea	Solid-state fermentation with *Bacillus subtilis* lwo (SSF)	Peptides with MW < 10 kDa produced after 12 h of fermentation (25.8 mg/g).	Antioxidant	0.5–1 mL of extract	Control groups (blank or without extract/salicylic acid)	Antioxidant activity increases with fermentation time.	[60]
Chickpea	*L. acidophilus* fermentation of pretreated dried chickpeas	Fermented chickpea protein peptides	Antidiabetic	5 mg/mL	Unfermented chickpea	*L. acidophilus*–fermented peptides suppressed α-glucosidase by >58%	[72]
Faba bean	Fermentation of faba bean flour with *L. plantarum* 299 v (30 °C, 3 d)	6 peptide sequences; most active fraction (3 kDa) contained di-/tripeptide motifs (e.g., GL, DA, MY)	Antihypertensive	NA	Control sample (without fermentation)	3.5–7.0 kDa peptides with higher ACE inhibitory activity (IC_50_ of 0.28 mg/mL);	[63]
Lentil	Red-lentil protein isolate fermented with multiple LAB and yeasts	*H. uvarum* SY1 led to the highest abundance of BAPs	Antioxidant/Antihypertensive	Different concentrations	Trolox; unfermented red lentil protein isolate	Antioxidant and ACE-inhibitory activities	[65]
Lupin	Solid-state fermentation with *L. plantarum* K779 (35 °C, 72 h)	Fermentation liberates peptides and phytochemicals	Antioxidant/Antihypertensive	20–200 µL	Non-inoculated samples	Enhanced antioxidant and ACE-inhibitory activity compared to raw	[66]
Pea	Fermentation of pea seeds with *L. plantarum* 299 v (22 °C, 7 d)	LC fractions yielded peptide KEDDEEEEQGEEE	Antihypertensive	NA	Sample without fermentation process	After fermentation + simulated digestion, ACE inhibition IC_50_ = 0.19 mg/mL (vs 0.37 control);	[64]
Quinoa	Solid-state fermentation with *L. plantarum* K779 (35 °C, 72 h)	Fermentation liberates peptides and phytochemicals	Antihypertensive	20–200 µL of sample	Non-inoculated samples	Enhanced antioxidant and ACE-inhibitory activity compared to raw	[66]
Quinoa	Solid-state fermentation of quinoa flour with *Lactobacillus paracasei* CICC 20241	5 potential ACE inhibitory peptides	Antihypertensive	0.05 and 0.1 mg/mL	ACE without peptides	NIFRPFAPEL: IC_50_ = 49.02 µM; AALEAPRILNL IC_50_ = 79.72 µM)	[69]
Red quinoa	Solid-state fermentation of *Chenopodium formosanum* sprouts (FCS) with *Rhizopus oligosporus*; (4 days, 35 °C with aeration	Glycine-rich peptides (GGGGGKP)	Antioxidant	0.05, 0.1 mg/mL	*In vitro* (Hs68 cells), 1 mmol/L N-acetylcysteine was used as a positive control; *In vivo*: untreated cells and worms (*Caenorhabditis elegans*)	GGGGGKP enhanced antioxidant defense and anti-aging effects by activating the Nrf2 pathway in cells and extending lifespan and stress resistance in *C. elegans*	[73]
Soy	Prozyme pretreated soy protein isolate fermented with *Lactobacillus rhamnosus* EBD1 (48 h at 37 °C)	Fermented soy protein hydrolysate	Antihypertensive/ Prebiotic	10–100 mg/kg hydrolysate per kg BW/day (6 weeks)	Spontaneously hypertensive rats (SHR) water or captopril	Rapid BP reduction, inhibited ACE, improved NO and SOD, remodeled gut microbiota (↓*Firmicutes*/*Bacteroidetes* ratio)	[74]
Soybean peptides	NA	Novel peptides identified	Prebiotic	NA	Undigested protein, MRS, FOS	Stimulated *L. reuteri* growth, unique fermentation profile	[75]

NA = information not available.

### 3.3. Comparative Perspective

**Figure 3:**
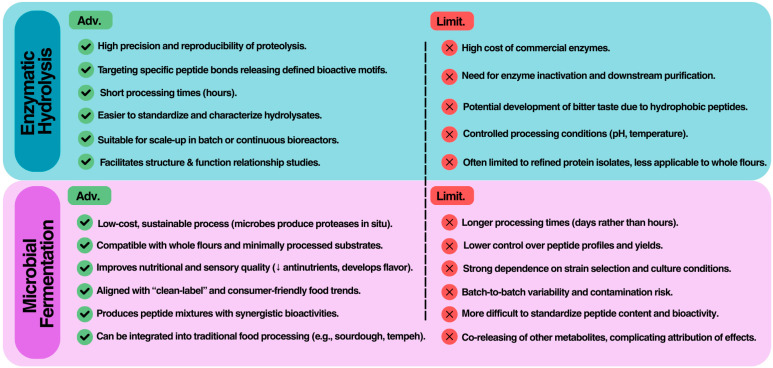
Practical advantages and limitations of enzymatic hydrolysis vs. microbial fermentation for the production of BAPs from pulses and pseudocereals.

When considered together, enzymatic hydrolysis and microbial fermentation should not be seen as competing but rather as complementary approaches for the production of BAPs (Figure 3). From a practical standpoint, enzymatic hydrolysis provides high reproducibility and control over proteolysis, making it particularly well suited for mechanistic studies, structure–activity relationship investigations, and the generation of purified peptides for multiple applications. Industrially, hydrolysis can be scaled through batch or continuous reactor systems, but costs remain heavily dependent on enzyme price, stability, and the need for inactivation and downstream purification. Furthermore, hydrolysates often require debittering due to the release of hydrophobic peptides, a factor that complicates direct use in food matrices.

Fermentation, in contrast, offers a low-cost and sustainable strategy, since microorganisms grow on inexpensive substrates and continuously generate proteolytic enzymes in situ. This approach is especially advantageous when the goal is to enrich whole flours or fermented foods with peptides, while simultaneously improving sensory and nutritional attributes (e.g., reduction in antinutritional factors, enhanced digestibility, and flavor development). Fermentation is also aligned with consumer expectations for natural and “clean-label” products. However, fermentation is less predictable than enzymatic hydrolysis; peptide yield and bioactivity depend strongly on strain selection, inoculum size, its metabolism, substrate composition, and environmental parameters such as pH, temperature, and moisture content. Moreover, batch-to-batch variability and the risk of contamination remain critical challenges in large-scale applications.

As reported in the previous sections, different processing methods lead to the generation of different peptides. The type of peptides produced, their structure, and their biological activities depend strongly on the hydrolysis method and purification approaches used. During peptide production, factors, such as the choice of enzyme, distinct peptide sequences and activities. For instance, fava bean proteins hydrolyzed with gastric enzymes produced peptides with anti-inflammatory capacity, while fermentation with *L. plantarum* produces peptide mixtures with ACE-inhibitory properties [36,64]. Pre-treatment processes such as pre-cooking, germination, or fermentation, also affect peptide release by altering protein structure or availability within the food matrix. In addition to production methods, the separation and purification of peptides lead to greater diversification of the final profiles. This, in turn, facilitates the selection of specific peptides based on the desired application.

A major challenge in the comparative evaluation of hydrolysis and fermentation lies in the lack of harmonized methodologies for peptide production and analysis. Differences in enzyme sources, hydrolysis conditions (e.g., E:S ratio, temperature, and time), microbial strains, and digestion models hinder direct comparison between studies and complicate meta-analytical interpretations of BAP efficacy. This methodological heterogeneity often leads to inconsistent peptide profiles and bioactivity outcomes, impeding regulatory acceptance and product standardization.

In terms of regulatory and market perspectives, enzymatic hydrolysates are closer to purified nutraceuticals, allowing more straightforward characterization, standardization, and dose definition. Fermented products, on the other hand, fit more readily into functional foods but typically deliver complex peptide mixtures whose exact contributions to health effects are more difficult to isolate. Overall, enzymatic hydrolysis is the superior method for producing bioactive peptides when the goal is a controlled, reproducible, and quantifiable bioactivity, supported by defined peptide structures and scalable standardization. Microbial fermentation, while advantageous for natural, sustainable food applications, remains less precise due to variability in microbial protease expression and substrate interactions.

A promising direction for future development is therefore the integration of both approaches, for example, by combining controlled enzymatic pretreatment with fermentation, in order to maximize peptide yield and multifunctionality while mitigating the drawbacks of each method.

## 4. Potential Bioactivities of BAPs

**Figure 4:**
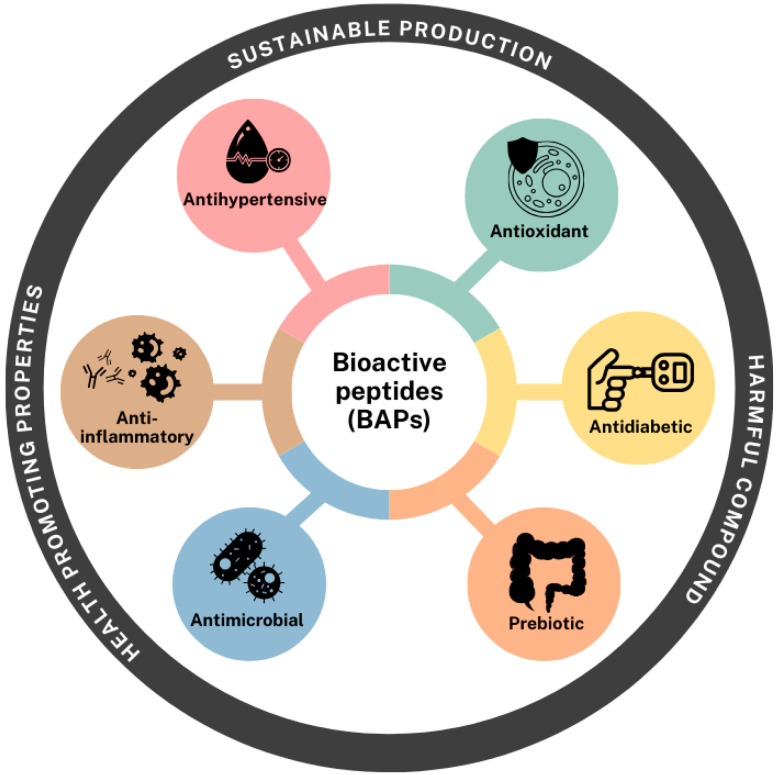
Potential bioactivities from pulses and pseudocereals BAPs.

Peptides obtained from pulses along with pseudocereals have demonstrated promising bioactivities through a wide range of mechanisms. Numerous studies have highlighted their protective roles against chronic and degenerative diseases, including cardiovascular disorders, type 2 diabetes, and inflammation-related conditions [76,77,78,79,80,81,82]. BAPs can influence metabolic pathways by modulating immune responses, improving insulin sensitivity, lowering blood pressure via ACE inhibition, and exerting antioxidant, antimicrobial or prebiotic effects (Figure 4) [78,83].

The following sections provide a comprehensive overview of the bioactivities investigated for hydrolysates and peptides produced through enzymatic techniques (Table 1) and microbial fermentation (Table 2).

### 4.1. Antioxidant Activity

Recent studies have increasingly highlighted the potent antioxidant potential of peptides derived from pulses and pseudocereals, revealing multiple underlying mechanisms through which they exert their bioactivity [14]. These peptides demonstrate the capacity to directly scavenge reactive oxygen species (ROS), chelate redox-active metal ions, and modulate intracellular antioxidant defense. What is particularly compelling is the role of specific amino acid residues. High activity is consistently linked to the presence of aromatic residues such as tryptophan (W), tyrosine (Y), phenylalanine (F), and histidine (H), which donate electrons or protons to stabilize free radicals; among these, W shows the strongest radical scavenging capacity, followed by Y and H. Hydrophobic amino acids including valine (V), leucine (L), alanine (A), isoleucine (I), proline (P), and methionine (M) increase solubility in lipid environments, enabling peptides to access radicals in the fat phase and donate protons effectively. Sequence location is also critical: residues such as V or I at the N-terminal and Y, W, or L at the C-terminal significantly enhance scavenging activity [17,84]. For instance, as reported in the study of Martineau-Côte et al. (2024) [85], the faba bean peptide VVIPTEPPHA exhibited stronger antioxidant activity measured by ABTS assay, than its shorter counterpart VIPTEPPHA due to an additional N-terminal V, while removal of the terminal L from TETWNPNHPEL markedly reduced radical scavenging. Paz et al. (2021) [86] demonstrated that the sulfur-containing amino acids, particularly cysteine (C), and methionine (M), obtained from *Amaranthus caudatus* L. enzymatic hydrolysis, contribute through their redox-active side chains. chains. In addition, acidic (E, D) and basic residues (H, R) improve electron donation and stabilize ROS [84,85,86,87].

In cellular systems (damage model of mouse skeletal muscle myoblast), peptides isolated from *Tartary* buckwheat have been shown to significantly reduce ROS accumulation and lipid peroxidation [88]. This protective effect seems to be linked to the stabilization of mitochondrial membrane potential and a concurrent rise in catalase activity, pointing toward preservation of mitochondrial integrity under oxidative stress. Similar protective responses have been observed in hepatocytes treated with novel pea-derived peptides, including YLVN, EEHLCFR, and TFY [89]. These peptides, obtained by a series of purification steps including ultrafiltration, ion exchange chromatography, G25 gel filtration chromatography, and reversed-phase chromatography, not only demonstrated strong *in vitro* DPPH and ABTS radical scavenging activity but also enhanced cell viability under H_2_O_2_-induced oxidative stress. Their antioxidant effects extended to the prevention of intracellular ROS accumulation and the upregulation of key enzymes involved in antioxidant defense, such as superoxide dismutase (SOD) and catalase (CAT).

Moreover, computational docking studies suggest that these peptides may activate the Keap1–Nrf2 signaling pathway, a central regulator of cellular redox homeostasis. By disrupting Keap1-mediated inhibition of Nrf2, the peptides potentially facilitate increased expression of downstream antioxidant enzymes like heme oxygenase-1 (HO-1), thereby amplifying endogenous cytoprotection mechanisms.

The antioxidant potential of these peptides has also been demonstrated *in vivo*. In hypertensive rat models, oral administration of a red quinoa protein hydrolysate led to a significant increase in tissue glutathione levels alongside a marked reduction in malondialdehyde (MDA), a lipid peroxidation marker [54]. Fermented red quinoa (*Chenopodium formosanum*) showed promising antioxidant potential and anti-aging properties due to the production of glycine-rich peptide. Produced through *Rhizopus oligosporus* fermentation, GGGGGKP demonstrated significant protective effects against UVA-induced oxidative stress in human dermal fibroblasts (Hs68), enhancing cell viability, activating the Nrf2 antioxidant pathway, and upregulating defense-related genes (Nrf2, HO-1, SOD, GSR) while downregulating senescence-associated markers such as p53, p21. Furthermore, it extended lifespan and improved oxidative stress resistance in *Caenorhabditis elegans* through activation of the SKN-1 pathway, the ortholog of human Nrf2. Collectively, these findings suggest that GGGGGKP represents a bioactive peptide with strong potential for application in functional foods or nutraceuticals aimed at mitigating oxidative damage and promoting healthy aging [73,90].

These outcomes reflect an enhanced systemic antioxidant status and further support the relevance of these peptides beyond cellular models.

Additional findings suggest that metal chelation is another critical aspect of the antioxidant function of legume-derived peptides. By binding redox-active metals such as iron and copper, these peptides may effectively inhibit reactions that generate harmful hydroxyl radicals. Collectively, the evidence points to a multifaceted antioxidant action, ranging from direct radical scavenging and metal chelation to the activation of redox-sensitive signaling pathways. Peptides derived from bean protein isolate and phaseolin have shown notable metal chelating activity following enzymatic hydrolysis [46]. Among the resulting size-fractionated peptides, those in the 0.7–1.0 kDa range exhibited the highest copper chelation (up to 82%), while iron binding was strongest in select isolate fractions, though generally lower. Phaseolin peptides appeared to be the main contributor to both antioxidant and copper chelating effects.

### 4.2. Anti-Inflammatory Effect

Inflammation is a physiological defense response of the immune system against infection, tissue damage, or toxic insults, aiming to eliminate pathogens and promote repair. While acute inflammation provides short-term protection, uncontrolled chronic inflammation is linked to the onset of serious disorders, including cardiovascular disease, diabetes, asthma, inflammatory bowel disease, and cancer. Key pro-inflammatory mediators such as nitric oxide (NO), tumor necrosis factor-α (TNF-α), and prostaglandin E_2_ (PGE_2_) are produced in activated immune cells, often through stimulation of the NF-κB pathway, which regulates cytokine and chemokine release. BAPs from legumes and pseudocereals have emerged as promising natural anti-inflammatory agents, with their activity strongly tied to specific amino acid residues and motifs [76].

The best-characterized example is the RGD motif, found in the soybean peptide lunasin (also detected in quinoa), which enables binding to the αVβ3 integrin and subsequent inhibition of NF-κB signaling [76]. Related motifs such as RGE have been identified in quinoa (chenopodin) and buckwheat (2S albumin), suggesting structural conservation in peptides with NF-κB inhibitory potential [14].

Additionally, hydrophobic (V, L, A, P, M), aromatic (W, Y, F), sulfur-containing (C, M), and basic (R, H) residues contribute to anti-inflammatory properties both directly, by interfering with inflammatory signaling, and indirectly, by reducing oxidative stress that drives NF-κB activation, as reported tin the previous Section.

Quinoa albumin fractions (rich in the anti-inflammatory lunasin, a 43-amino-acid peptide [91]) reduced IL-1β-induced NF-κB/IL-8 expression by up to 74% [14]. Importantly, in the same study, simulated gastrointestinal digestion appears to enhance these effects; digests of quinoa and buckwheat protein fractions and peptides significantly reduced IL-1β–driven cytokine release in Caco-2 cells. Collectively, the evidence indicates that digestion-released peptides, with low-molecular-weight fragments (≤14 kDa), may exert cytoprotective effects by mitigating NF-κB–driven transcriptional activation in response to inflammatory stimuli.

A recent study on the Panhandle Pride white bean cultivar, developed by the University of Nebraska, demonstrated that the <3 kDa peptide fraction markedly attenuates TNF-α–induced inflammatory and oxidative responses in human vascular endothelial cells [92]. Among all tested molecular weight fractions, only the <3 kDa filtrate significantly suppressed TNF-α–driven expression of key inflammatory biomarkers, including intracellular adhesion molecule-1 (ICAM-1), vascular adhesion molecule-1 (VCAM-1), and the chemokine MCP-1. This fraction, enriched in γ-glutamyl peptides and hydrophobic dipeptides, retained its bioactivity following simulated gastrointestinal digestion. Notably, the digested filtrate continued to downregulate VCAM-1, E-selectin, and MCP-1 expression at peptide concentrations as low as 250 μg/mL.

Comparable anti-inflammatory potential has been observed in lupin protein hydrolysate. Using a Caco-2/THP-1 macrophage co-culture model to simulate intestinal absorption, the hydrolysate at 0.1–0.5 mg/mL effectively counteracted LPS-induced upregulation of pro-inflammatory cytokine mRNA (TNF-α, IL-1β, IL-6), restoring expression levels to those of unstimulated controls [49]. Notably, IL-1β mRNA levels were reduced below baseline, indicating a pronounced anti-inflammatory effect.

The immunomodulatory properties of lupin protein hydrolysate have also been confirmed *in vivo*. In a murine model of experimental autoimmune encephalomyelitis, an established model of multiple sclerosis characterized by elevated pro-inflammatory cytokine production during the acute phase, prophylactic administration of lupin protein hydrolysate at 100 mg/kg significantly attenuated disease severity. Treated mice maintained higher functional performance scores relative to controls, exhibiting milder neurological deficits such as unilateral hind-limb paralysis or impaired righting reflex [50].

### 4.3. Antihypertensive Effect

Many legumes and pseudocereal-derived peptides lower blood pressure by targeting the renin–angiotensin system and enhancing vasodilatory pathways. The mechanism of ACE inhibition by BAPs is based on their ability to interact with the enzyme’s active site and block the conversion of angiotensin I into the vasoconstrictor angiotensin II and preserving the vasodilator bradykinin. ACE is a zinc metalloprotease, and effective ACE-inhibitory peptides are typically short sequences (often di- or tripeptides) that can fit into the active site with high affinity. Their inhibitory activity relies on several structural features: (i) functional groups capable of coordinating with the catalytic zinc(II) ion; (ii) side chains able to establish hydrogen bonds with polar subsites within the enzyme; (iii) proline (P)-containing motifs that interact with the proline-specific pocket, enhancing stability and resistance to degradation; and (iv) hydrophobic or aromatic residues such as phenylalanine (F), tryptophan (W), and tyrosine (Y) that bind preferentially to the hydrophobic pocket of ACE. The positioning of these residues, particularly at the C-terminal end, is crucial for activity, with sequences ending in P, W, Y, or arginine (R) frequently reported as the most potent inhibitors [93].

*In vitro* studies have identified many ACE-inhibitory sequences encrypted in these proteins. For example, a novel pentapeptide SAPPP was purified from quinoa bran globulin hydrolysate with an ACE-inhibitory IC_50_ of 915 μM, while the activity of SAPPP after the digestion was calculated to be 926 μM [52]. In spontaneously hypertensive rats, orally administered pulse-derived peptides have shown clear antihypertensive effects [56]. A tetrapeptide from enzymatically treated soymilk, FFYY, given at only 80 µg/kg body weight per day, significantly attenuated the rise in systolic and diastolic blood pressure in SHR over 3 weeks, without adverse effects. The tetrapeptide showed strong inhibitory activity to the ACE-enzyme with an inhibitory concentration of 1.9 µM. Similarly, Daliri et al. (2020) demonstrated that a single oral dose of a fermented soybean protein hydrolysate produced a rapid antihypertensive effect in SHR [74]. Systolic blood pressure fell by 25 mmHg and diastolic by 40 mmHg within 6 h of the hydrolysate administration, comparable to the ACE inhibitor drug captopril. Mechanistically, fermented soybean protein hydrolysate feeding acutely inhibited serum ACE activity in the rats while elevating vasoprotective mediators (increased nitric oxide and superoxide dismutase levels, reduced malondialdehyde). Notably, the soy peptide treatment remodeled the gut microbiome of hypertensive rats, suggesting a gut-mediated contribution to its antihypertensive action (see Section 4.6).

Quinoa proteins have likewise yielded antihypertensive peptides. *In vitro*, quinoa protein hydrolysates obtained through high pressure-assisted enzymatic hydrolysis displayed strong ACE-inhibitory activity [53]. The <3 kDa permeate, characterized by a higher degree of hydrolysis, was particularly effective at concentrations of 0.1 and 0.5 mg/mL, showing greater inhibition than either non-hydrolyzed proteins or hydrolysates produced under atmospheric pressure. This fraction contained several peptides with potent ACE-inhibitory potential, among which GSHWPFGGK, FSIAWPR, and PWLNFK exhibited the highest PeptideRanker scores.

Similarly, amaranth proteins have been shown to release potent antihypertensive peptides upon fermentation. Hydrolysates from *Amaranthus hypochondriacus* seeds fermented with *Enterococcus faecium* LR9 displayed the strongest ACE inhibitory activity (79%) at a concentration of 0.00625 mg/mL, outperforming those obtained with *Leuconostoc mesenteroides* 18C6 (68%) and enzymatic hydrolysis with Alcalase (69%) [71]. Peptide identification by tandem mass spectrometry revealed 125 sequences derived mainly from agglutinin, cystatin, 11S globulin, and trypsin inhibitor. Notably, the highest degree of proteolysis did not correspond to the strongest inhibition, suggesting that specific peptide release, rather than overall hydrolysis, determines ACE inhibitory capacity.

### 4.4. Antidiabetic Effect

A key mechanism in antidiabetic effect is inhibition of dipeptidyl peptidase-IV (DPP-IV), the enzyme that degrades incretin hormones like GLP-1 [94]. By inhibiting DPP-IV, food-derived peptides prolong endogenous GLP-1 action, thereby enhancing insulin secretion and improving glycemic control. Inhibition of digestive enzymes such as α-glucosidase and α-amylase represents another antidiabetic mechanism of legume-derived peptides. Several studies have identified potent DPP-IV, α-glucosidase and α-amylase inhibitory peptides from legume and pseudocereal proteins, that are typically short, proline-rich, and hydrophobic, with activity strongly influenced by residues at the N- and C-termini. Proline or alanine at the N-terminal second position, often combined with hydrophobic residues such as I, L, V, or M, enhances binding to the enzyme’s active site, as shown in sequences like IPM and LPVPQ in milk protein. QSAR and molecular docking further confirm that cysteine-containing dipeptides also contribute to inhibition [17].

Lupin-derived peptides have garnered attention for multi-faceted antidiabetic effects. A recent study characterized an Alcalase-generated lupin protein hydrolysate (LPH) and demonstrated its anti-diabetic activity via DPP-IV modulation [47]. Although lupin protein consumption was previously known to improve glucose metabolism, direct evidence of DPP-IV inhibition was lacking. The study confirmed that LPH potently inhibits DPP-IV *in vitro* and in cultured cells, leading to prolonged GLP-1 activity. The authors demonstrated that more than 140 peptides derived from lupin protein hydrolysate (LPH) are capable of trans-epithelial intestinal transport. The bioactivity of these peptides was further validated *in vivo*, where LPH supplementation inhibited DPP-IV activity and exerted glucose-lowering effects both in mice (100 mg/kg) and in humans following a 28-day intervention with a beverage containing 1 g of hydrolysate per 200 mL serving.

Chickpea peptides, in particular, display strong α-glucosidase inhibitory activity. Ma et al. optimized a fermentation-based method to produce chickpea peptides with enhanced antidiabetic activity [72]. The fermentation with microbial strains generated low-molecular-weight fractions that suppressed yeast α-glucosidase activity by over 58% at 5 mg/mL, with *Lactobacillus acidophilus*–fermented chickpea producing the most potent inhibitors.

Soybean-derived peptides exhibit dual enzyme inhibition [55]. Germinated soybean produced 5–10 kDa fractions with strong DPP-IV inhibitory activity (IC_50_ 0.91 mg/mL), alongside significant inhibition of salivary α-amylase and intestinal α-glucosidases. The active sequences, ranging from 6 to 18 amino acids, were predominantly derived from β-conglycinin, glycinin, and P34 probable thiol protease.

Beyond digestive enzyme inhibition, certain pulse-derived peptides modulate glucose metabolism through effects on insulin signaling and hepatic pathways [48]. Hydrolyzed γ-conglutin (Cgh) from Andean lupin, originates from the Andean region of Ecuador, completely inhibited DPP-IV activity at 5 mg/mL and enhanced glucose uptake 6.5-fold in an enterocyte/adipocyte co-culture by promoting GLUT-4 translocation. Additionally, in an enterocyte/hepatocyte system, Cgh reduced gluconeogenesis by 50% and downregulated phosphoenolpyruvate carboxykinase expression.

### 4.5. Antimicrobial Capacity

Antimicrobial peptides (AMPs) are generally classified into four main subgroups based on charge and structure: anionic, cationic α-helical, cationic β-sheet, and extended cationic peptides. Anionic antimicrobial peptides often interact with microbial membranes through metal ion–mediated salt bridges. Cationic α-helical peptides are amphipathic and disrupt membranes after adopting α-helical conformations. Cationic β-sheet antimicrobial peptides are stabilized by intramolecular disulfide bonds; their bactericidal action relies on electrostatic binding to negatively charged microbial surfaces followed by membrane permeabilization. Extended cationic AMPs, rich in residues such as proline, arginine, or tryptophan, lack defined secondary structures but insert into and destabilize membranes through hydrophobic and electrostatic interactions. Indeed, several studies in the past few years have isolated plant defense peptides or generated hydrolysates from pulses that are active against bacteria. Hydrolysates of adzuki bean and mung bean prepared with peptidase from *Aspergillus oryzae* showed fraction-dependent antibacterial activity against major foodborne pathogens [44]. Hydrolysate fraction from adzuki bean achieved 76% inhibition of *Salmonella typhimurium*, while mung bean peptides fraction inhibited 71% of *Staphylococcus aureus* at 2 mg/ mL, effects comparable to gentamicin. LC–MS/MS identified short, cationic peptides, with lysine-rich sequences most effective against *S. aureus* and proline motifs linked to *Salmonella* inhibition.

Osman et al. produced an Alcalase hydrolysate of cowpea seed proteins and fractionated it by size, finding strong antimicrobial action against both Gram-positive and Gram-negative pathogens [45]. MIC for cowpea peptides (in the <1 kDa fraction) was as low as 25 µg/mL against *E. coli*, *P. aeruginosa*, and *S. pyogenes*, while 100–150 µg/mL was needed against *S. aureus* and *Listeria* (which are Gram-positive and generally more peptide-resistant). Even at those levels, the cowpea hydrolysate was highly effective, reducing bacterial loads substantially. Electron microscopy of treated bacteria revealed membrane damage and leakage, confirming a lytic mode of action. Mass spectrometry analysis identified multiple small di-peptides (180–370 Da) and one penta-peptide (659 Da) in the active cowpea fraction. Among the ten peptides identified in positive ion mode, three contained arginine residues, classifying them as cationic peptides with net positive charge. Similarly, two of the five peptides detected in negative ion mode also carried arginine, confirming their cationic nature.

Complementarily and beyond growth inhibition, a red kidney bean peptide extract at the MIC (1.25 mg/mL) suppressed biofilm formation by up to 87% and markedly reduced quorum sensing–regulated virulence factors in *P. aeruginosa* (pyocyanin and pyoverdine) [95]. These findings suggest a dual mechanism: direct antimicrobial effects and anti-virulence activity that may limit resistance development.

### 4.6. Prebiotic Effect

Prebiotics were long regarded solely as non-digestible carbohydrates that stimulate beneficial gut microbes. The latest consensus from the International Scientific Association for Probiotics and Prebiotics (ISAPP), however, defines them as “*a substrate that is selectively utilized by host microorganisms conferring a health benefit.*” [96]. This broader definition opens the possibility for non-carbohydrate compounds, including peptides, to be considered prebiotics when they modulate the gut microbiota in a health-promoting manner. As noted above, fermented soy protein hydrolysate (P-SPI) not only lowered blood pressure in SHR but also markedly improved gut microbiota diversity and balance. In Daliri et al.’s experiment, in hypertensive rats, fermented soy protein hydrolysates restored microbial diversity and reduced the *Firmicutes/Bacteroidetes* ratio, a dysbiosis marker associated with hypertension. Chronic supplementation enriches short-chain fatty acid (e.g., propionate) producing genera while decreasing pro-inflammatory taxa such as *Streptococcaceae* and *Erysipelotrichales*.

Other studies echo these findings. After simulated gastrointestinal digestion, soybean-derived peptides (DPPs) stimulated the growth of *Lactobacillus reuteri* more effectively than non-digested soybean proteins, undigested peptides, or the MRS control medium [75]. Metabolic activity was further assessed by monitoring lactic acid production. Interestingly, lactic acid levels in the FOS and FOS + DPP groups were significantly lower than those in the DPP-only group after 8 h of incubation and remained stable thereafter, suggesting distinct fermentation dynamics when peptides are combined with carbohydrates. Peptide profiling revealed several novel sequences (ILSPL, IQLP, IAANPA, FASPA, IATSPA, and IIP), with most characterized by a proline residue at the penultimate position and isoleucine at the N-terminus

More direct evidence of prebiotic effects comes from *in vivo* studies. In a high-fat diet (HFD) mouse model, lupin protein hydrolysate supplementation for 12 weeks reduced weight gain, improved adipose function, and counteracted dyslipidemia and insulin resistance [51]. These effects were associated with microbiota modulation: LPH restored the abundances of specific taxa (*Patescibacteria*, *Thermodesulfobacteriota*) and increased beneficial *Verrucomicrobiota*, notably *Akkermansia*, whose abundance, reduced under HFD, was fully recovered. *Akkermansia muciniphila* is a key species linked to improved metabolic health and healthy balanced gut biodiversity.

## 5. Regulatory Hurdles: Peptides as Novel Foods

**Figure 5:**
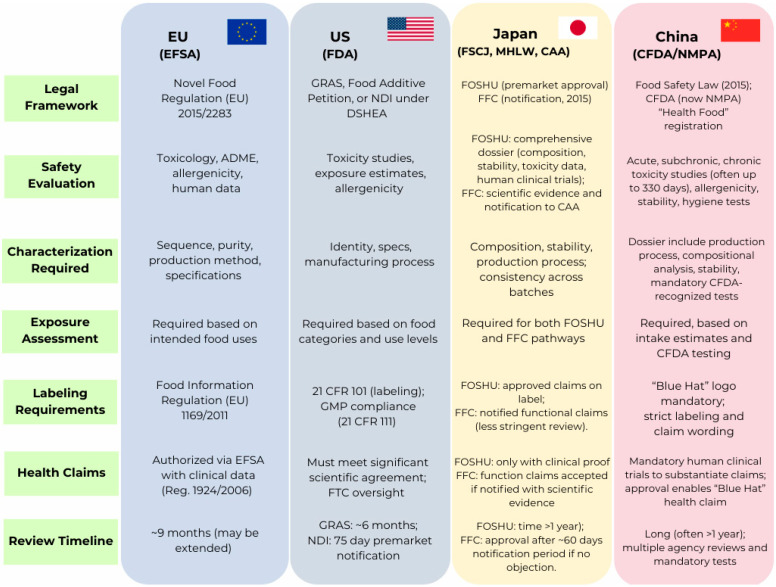
Comparative overview of regulatory frameworks governing the approval of bioactive peptides as foods or health products in the European Union (EFSA), United States (FDA), Japan (FSCJ, MHLW, CAA), and China (CFDA/NMPA). The figure summarizes requirements for safety evaluation, product characterization, exposure assessment, labeling, health claims, and typical review timelines across jurisdictions.

Despite the increasing interest in BAPs for their functional and health-promoting properties, their incorporation into functional foods, nutraceuticals, or supplements is subject to stringent regulatory scrutiny. European Union (EU), United States (US), Japan and China have established frameworks to ensure the safety and proper labeling of such ingredients before market authorization. This section outlines the key regulatory requirements imposed by the European Food Safety Authority (EFSA), US Food and Drug Administration (FDA), the Food Safety Commission of Japan (FSCJ), and China Food and Drug Administration (CFDA) for peptides intended as novel food ingredients or dietary supplements. Despite procedural differences, the EFSA, FDA, FSCJ, and CFDA emphasize comprehensive safety assessments, proper characterization of peptide ingredients, and substantiation of health-related claims [97,98,99]. Figure 5 summarizes the core regulatory requirements for peptide-based novel foods in the EU, US, Japan, and China.

This comparison highlights the regulatory hurdles that must be overcome before BAPs can be successfully integrated into food systems or marketed as dietary supplements. While regulatory alignment between jurisdictions is improving, scientific substantiation of safety and efficacy remains the cornerstone of market authorization for BAPs.

### 5.1. European Regulatory Framework

In the EU, BAPs are regulated under Regulation (EU) 2015/2283 on novel foods, which governs food ingredients not consumed to a significant extent in the EU before 15 May 1997. Additional requirements apply under Regulation (EC) 1924/2006 on nutrition and health claims made on foods if the peptide ingredient is also intended to make health claims [97,98,100,101].

EFSA requires a comprehensive safety assessment as part of the novel food dossier. This includes molecular characterization of the peptides—such as amino acid sequence, molecular weight distribution, degree of hydrolysis, and purity profile—along with a full description of the source material, enzymatic hydrolysis process, and downstream purification steps [101]. Applicants must also provide a detailed compositional analysis, including the peptide profile, residual proteins, potential contaminants (e.g., heavy metals, microbial toxins), and batch-to-batch consistency. Stability data during storage and within the intended food matrix must demonstrate that the peptide retains its integrity and function under real-world conditions [101].

The application is required to define the target food categories, the intended use levels, and the estimated dietary exposure, with these definitions to be based on population intake data. EFSA’s customary approach involves the evaluation of both mean and high-percentile (95th) exposure scenarios [101].

Safety assessments typically involve *in vitro* and *in vivo* toxicology studies, including genotoxicity, subchronic (90-day) toxicity, and, if required, reproductive or developmental studies. Human clinical data, while not mandatory, are often beneficial to support safety and efficacy [101,102]. EFSA also evaluates whether the peptide has any adverse nutritional implications when added to the diet. Given that many BAPs are derived from allergenic sources (e.g., milk, soy, fish), applicants must assess potential allergenicity using *in silico* sequence homology tools, *in vitro* digestibility tests, and, where appropriate, allergen-specific immunoassays [101,102].

If a health claim is proposed (e.g., blood pressure regulation or improved satiety), it must be separately substantiated with human intervention trials in accordance with Regulation (EC) 1924/2006 [100,102]. EFSA requires that these studies demonstrate a clear cause–effect relationship between peptide consumption and the claimed physiological benefit. Once EFSA issues a favorable scientific opinion, the European Commission can authorize the peptide for use as a novel food through an implementing act published in the EU Official Journal [103].

### 5.2. United States Regulatory Framework

In the US, regulatory oversight of food-derived peptides depends on their intended use. Peptides incorporated into conventional foods must either be generally recognized as safe (GRAS) under the Federal Food, Drug, and Cosmetic Act, approved as food additives, or subject to a New Dietary Ingredient (NDI) Notification if intended for use in dietary supplements under the Dietary Supplement Health and Education Act (DSHEA, 1994) [104,105].

GRAS notification is the most common regulatory route. Applicants must demonstrate that the peptide ingredient is safe under its intended conditions of use, based on publicly available scientific evidence and, in most cases, the consensus of qualified experts. The submission includes detailed information on identity, manufacturing process, and specifications, including the source, production (e.g., enzymatic hydrolysis), purification, and testing for contaminants or residual solvents [104]. Use levels across different food categories and corresponding estimated daily intake based on population data must be provided. Toxicological evaluations, similar to EFSA requirements, involve genotoxicity, subchronic, and—if warranted—chronic toxicity studies, along with assessments of allergenicity and absorption/metabolism data [104,106,107].

For dietary supplements, if the peptide ingredient was not marketed in the U.S. before 15 October 1994, it qualifies as a New Dietary Ingredient (NDI) and must undergo a premarket safety notification at least 75 days before marketing. The notification includes safety evidence, manufacturing details, and labeling information. Supplement formulations must also comply with Good Manufacturing Practices (GMP) outlined in 21 CFR Part 111 [104,106,107]. Labeling and claims are regulated under 21 CFR Part 101. While structure/function claims (e.g., “supports healthy blood pressure”) are permitted with a mandatory disclaimer, health claims linking the peptide to disease risk reduction require FDA pre-approval based on significant scientific agreement [104,106,107].

### 5.3. Japanese Regulatory Framework

In Japan, oversight of BAPs in foods is provided by the Food Safety Commission of Japan (FSCJ) in collaboration with the Ministry of Health, Labour and Welfare (MHLW) and the Consumer Affairs Agency (CAA). Japan has a dual regulatory pathway for functional foods. The Foods for Specified Health Uses (FOSHU) system grants approval to individual products containing functional ingredients proven to regulate or support physiological functions, such as blood pressure or gastrointestinal health. It requires extensive premarket evaluation, including toxicological data, human clinical trials on Japanese subjects, and detailed compositional and stability information, before authorization is granted for health claims and often require publications in local journals. This process is rigorous and time-intensive, often exceeding one year. Once approved, the product can display the official FOSHU mark, which helps consumers identify products that have passed the rigorous government evaluation [108,109,110].

In contrast, the Foods with Function Claims (FFC) pathway, introduced in 2015, allows companies to notify the CAA of scientific evidence supporting a functional claim at least 60 days before marketing, without undergoing the same level of governmental review as FOSHU. For both systems, manufacturers must provide compositional data, manufacturing details, and safety assessments consistent with FSCJ guidance, emphasizing consumer safety and scientific substantiation [109].

### 5.4. Chinese Regulatory Framework

China has also established a dynamic regulatory framework for foods with health benefits, including BAPs and protein hydrolysates, under the authority of the China Food and Drug Administration (CFDA) and guided by the Food Safety Law (FSL) of 2015 [108,109]. BAPs are typically considered within the health food (HE) category, which includes dietary supplements and functional foods that are intended to regulate human body functions without treating diseases. Key hurdles include classification as “special foods,” stringent requirements for human clinical evidence to substantiate health claims, and mandatory testing conducted by CFDA-recognized agencies. These tests cover toxicological safety (acute, subchronic, and chronic toxicity studies often lasting up to 330 days), efficacy screening, hygiene standards, stability testing, and detailed production process documentation and also site visits to overseas production facilities to inspect processes and take samples Approval confers the right to use the “Blue Hat” logo, a symbol of CFDA-certified health foods [108,109,110]. However, the framework is described as being in flux, with evolving policies and guidelines that require continuous monitoring. Overall, the combination of mandatory long-term testing, dossier preparation, and regulatory uncertainty constitutes a major barrier to market entry for novel peptide-based foods in China.

## 6. Challenges Limiting the Commercial Development of BAPs

Despite the increasing recognition of food-derived BAPs as functional components with potential health-promoting effects, their commercial development faces substantial scientific, technological, and regulatory challenges. These limitations hinder both industrial scalability and the translation of promising laboratory findings into consumer-ready products [111].

One of the primary technical obstacles lies in the preparation and purification processes. Conventional extraction and purification methods, such as solvent extraction, ultrafiltration, and chromatography, are often labor-intensive, time-consuming, and result in low peptide yields. High-purity peptides (≥99%) are frequently targeted in commercial applications, especially for therapeutic or high-value nutraceutical products; however, achieving such purity substantially increases production costs. Moreover, ultra-purification may remove other bioactive constituents that contribute synergistically to the desired health effects [99,112].

Membrane filtration systems, although scalable, are prone to operational issues such as pore blockage, reduced selectivity, and poor reproducibility across batches. Chromatographic techniques, while precise, are economically prohibitive for industrial use—accounting for up to 70% of total capital and operational expenditures in peptide bioprocessing—thus limiting their feasibility beyond laboratory-scale production [112,113].

Processing conditions further influence the integrity and efficacy of BAPs. High-temperature treatments used to denature protein precursors can negatively affect peptide profiles, leading to chemical modifications such as racemization, cyclization, and the formation of advanced glycation end-products (AGEs) through Maillard reactions. These alterations not only reduce bioactivity but can also result in undesirable sensory changes and the formation of potentially toxic intermediates.

Organoleptic properties also present a barrier to consumer acceptance. Many peptide hydrolysates exhibit a bitter taste, attributed to hydrophobic amino acid sequences, which limits their applicability in food and nutraceutical formulations [114]. Processing strategies, especially enzyme selection, use of masking agents or sequential hydrolysis, can help in reducing bitterness and improve sensory qualities, supporting broader use in food products. However, these techniques frequently incur substantial expenses and may inadvertently diminish the peptide’s functional activity through structural modification or partial degradation [115,116].

Another considerable challenge is the low bioavailability of BAPs. Although many peptides demonstrate potent biological activities *in vitro*, these effects often fail to replicate *in vivo* due to enzymatic degradation during gastrointestinal transit. BAPs demonstrate a high degree of susceptibility to hydrolysis by proteolytic enzymes, including pepsin, trypsin, chymotrypsin, brush border peptidases and serum proteases. This process results in the generation of inactive fragments and a significant reduction in systemic availability [3,112]. Additional factors influencing poor bioavailability include high molecular weight, the presence of hydrophobic or basic amino acids at the C-terminal end (e.g., lysine, arginine), and inherently low intestinal permeability—all of which collectively impair absorption and subsequent bioactivity [112].

Furthermore, BAPs are commonly obtained as complex peptide mixtures, making it difficult to ensure batch-to-batch consistency and accurate characterization. Unlike single-entity pharmaceutical compounds, these mixtures challenge standardization, stability testing, and quality assurance protocols, which are essential for regulatory compliance and product reproducibility

Lastly, the lack of robust clinical trial data remains a critical limitation. Although numerous *in vitro* and animal model studies provide preliminary evidence of health benefits, these findings cannot reliably predict human outcomes due to differences in absorption, distribution, metabolism, and excretion (ADME) pathways [112]. Regulatory approval for functional foods or health claims typically necessitates comprehensive human intervention studies, including clear identification of active doses, biomarkers, and long-term safety data. Main regulatory organs rejections of health claims for BAPs typically cite insufficient evidence and methodological shortcomings in the submitted dossiers.

## 7. Concluding Remarks

This review provided an overview of recent advances in the production and characterization of BAPs derived from pulses and pseudocereals, with particular emphasis on bio-based approaches such as enzymatic hydrolysis and microbial fermentation. These high-quality plant proteins are a sustainable raw material for producing multifunctional peptides with antioxidant, antihypertensive, antidiabetic, antimicrobial and prebiotic properties. In this context, these bio-based approaches transform affordable, protein-rich crops into added-value functional ingredients for the food and nutraceutical industries.

By encompassing studies published in recent years, this review highlights both the technological strategies and biological evidence supporting the potential of pulse- and pseudocereal-derived BAPs. Enzymatic hydrolysis remains the most reproducible method for targeted peptide release, whereas microbial fermentation addresses the growing consumer demand for natural and “clean-label” products, providing additional nutritional and sensory benefits. Increasingly, these approaches are being integrated with *in silico* prediction tools, which accelerate peptide discovery and optimization for BAP production.

Looking ahead, the integration of computational prediction with fermentation-based approaches represents a promising yet underexplored research frontier. AI-based and *in silico* modeling could enable the identification of microbial proteolytic patterns, the design of strain-specific metabolic pathways, and the development of tailored fermentation strategies aimed at the targeted release of peptides with desired bioactivities. Despite these promising advances, key challenges remain regarding process standardization, large-scale production, and regulatory validation. Future research should also focus on deepening the understanding of structure–activity relationships and on validating the long-term health effects of BAPs through clinical trials.

The integration of BAPs into the global food and nutraceutical markets is promising but is heavily dependent on regulatory clearance. EU, US, Japan, and China all require rigorous demonstration of safety, quality, and efficacy, necessitating advanced analytical, toxicological, and clinical data. While these regulatory frameworks present certain challenges, they also ensure consumer protection and product credibility. Aligning research and development practices with these regulatory expectations not only accelerates approval timelines but also fosters trust among consumers and health professionals. In order to successfully commercialize BAPs, harmonization of global regulatory requirements and investment in standardized methodologies for the evaluation of the potential health benefits will be critical to the successful commercialization of BAPs.

Overall, the valorization of pulses and pseudocereals as sustainable protein sources for BAPs production not only expands opportunities for developing next-generation functional foods and nutraceuticals but also contributes to global strategies promoting sustainable diets and health-oriented innovation.

Collectively, these multifaceted challenges underscore the necessity for integrated approaches that combine advanced processing technologies, *in vivo* validation strategies, and regulatory harmonization. Such an approach is required to facilitate the translation of BAPs from bench to market.

## Data Availability

No new data were created or analyzed in this study. Data sharing is not applicable to this article.

## Abbreviations

The following abbreviations are used in this manuscript:

ACE	Angiotensin-I-converting enzyme
ADMET	Absorption, Distribution, Metabolism, Excretion, and Toxicity
BAP	Bioactive peptide
BP	Blood pressure
BW	Body weight
Cgh	Hydrolyzed γ-conglutin
DPP-IV	Dipeptidyl peptidase-IV
E:S	Enzyme-substrate ratio
EAA	Essential amino acid
EAE	Experimental autoimmune encephalomyelitis
HFD	High-fat diet mice
IC_50_	Half-maximal Inhibitory Concentration
ICAM-1	Intercellular Adhesion Molecule 1
IL-1β	Interleukin 1β
IL-6	Interleukin 6
IL-8	Interleukin 8
LAB	Lactic acid bacteria
LPS	Lipopolysaccharide
MD	Molecular dynamics
MM-GBSA	Molecular mechanics generalized Born surface area
MM-PBSA	Molecular mechanics Poisson–Boltzmann surface area
NF-κB	Nuclear factor kappa-light-chain-enhancer of activated B cells
QSAR	Quantitative structure–activity relationship
RMSD	Root-mean-square deviation
SHR	Spontaneously hypertensive rats
SOD	Superoxide dismutase
TE	Tolox equivalents
TNF-α	Tumor necrosis factor α
VCAM-1	Vascular Cell Adhesion Molecule 1
